# Paediatric rheumatology in Nigeria: history, challenges and the future

**DOI:** 10.3389/fped.2024.1440625

**Published:** 2024-10-03

**Authors:** Ayodele Faleye

**Affiliations:** Pediatric Rheumatology Unit, Department of Pediatrics, Lagos State University Teaching Hospital, Ikeja, Nigeria

**Keywords:** pediatrics, rheumatology, patients, rheumatic, childhood

## Abstract

Pediatric rheumatology is a relatively new subspecialty in Nigeria. For many years, rheumatic disease had not been well-recognized or studied in Nigeria, so there has been a low suspicion index. The clinical presentation of most pediatric rheumatic diseases (PRDs) mimics non-rheumatic diseases making it a diagnostic challenge most of the time. The story has changed in Lagos since the creation of a pediatric rheumatology unit at the Lagos State University Teaching Hospital. There is now a high index of suspicion for these diseases in the hospital but no one knows about what is happening in the other 35 states of the country who do not have a pediatric rheumatologist. Major challenges being faced presently are poor awareness among healthcare workers and the community, high cost of investigations and medications, lack of pediatric rheumatologists in most hospitals in the country, lack of pediatric rheumatology training centers for both undergraduate and postgraduate training, and lack of research data. The healthcare community in Nigeria is looking forward to a future where pediatric rheumatology will be highly recognized and established.

## Introduction

Pediatric rheumatology which is the study of rheumatic diseases in children and adolescents, had its origins in the first half of the 20th century, principally as a study of chronic inflammatory arthritis, the most common of the childhood rheumatic diseases (1). It is a relatively young subspecialty in Nigeria and much of Sub-Saharan Africa.

Rheumatic diseases are a group of chronic disorders characterized by symptoms involving the muscles, joints, bursae and tendons. They are mostly multisystem inflammatory diseases of the connective tissues with autoimmune markers (2). The diseases traditionally considered as pediatric rheumatic include rheumatic fever, juvenile idiopathic arthritis, the spondyloarthropathies, juvenile systemic lupus erythematosus (JSLE), juvenile dermatomyositis, juvenile scleroderma, vasculitis, and some rare disorders (3). All these conditions are characterized by prominent involvement of the musculoskeletal system and chronic or recurrent inflammation of connective tissues (3). These diseases may cause severe chronic pain, joint damage, increasing disability, and even death (4). An estimated 6–7 million children worldwide have rheumatic diseases, many of whom live in Africa and Asia (5, 6). Unfortunately, there is a paucity of pediatric rheumatologists worldwide, but worse in Africa and Asia (5). According to Henrickson et al. (5), while the number of pediatric rheumatologists-to-population is 0.008 per million in Africa and 0.04 per million in Asia, it is 2.4 per million in Europe and 3–4 per million in North America. Therefore, training healthcare workers who are capable of offering clinical care to pediatric rheumatology patients and improving their performance for this noble task is a critical global need but more so in Africa (5, 7).

### Overview of Nigeria as a context for healthcare and rheumatological services

Nigeria is Africa's most populous country, with an estimated population of 216 million people out of which 110 million are children. It is often referred to as the “Giant of Africa” owing to its large mostly young population and economy (8, 9). Lagos is Nigeria's largest city with an estimated population of over 25 million people (10). Nigeria has a decentralized healthcare system with three tiers of care: primary, secondary, and tertiary under the responsibilities of the Federal, State, and Local governments, respectively. The Local government manages the Primary Health Care facilities which are designed to be the closest to the people, offering a wide range of preventive, basic curative, and limited rehabilitative services. The State governments through their respective Ministries of Health manage General Hospitals (secondary healthcare) which have a limited range of specialist services and serve as referral facilities for public and private primary healthcare facilities. The apex of Nigeria's healthcare services are the tertiary care facilities comprising Teaching Hospitals, and Federal Medical Centers which have a wide range of specialist and sub-specialist expertise and services. The largest provider of healthcare services in Nigeria are the private hospitals, which offer mainly primary healthcare with few offering limited specialist services. Currently, most rheumatological services are domiciled in the public Teaching Hospitals.

Healthcare financing is majorly out-of-pocket payment as the National Health Insurance Scheme (NHIS) covers less than 5% of the populace, mainly the formal sector.

## History

In Nigeria, a formal history of pediatric rheumatology as a recognized subspecialty may be dated to 2018, though it was known for years that there were Nigerian children with pediatric rheumatic diseases (PRDs), even if not always widely recognized (11–20). The Department of Pediatrics of the Lagos State University Teaching Hospital (LASUTH), Ikeja, Lagos recognized the need for a Pediatric Rheumatology sub-specialty training program, and thereafter partnered with the well-established adult Rheumatology unit of the Department of Internal Medicine of the hospital to pioneer and evolve a specialist training in Pediatric Rheumatology.

It is worthy of note that since its inception in 2010 and until recently, the adult Rheumatology Clinic has been offering pediatric rheumatological services in conjunction with consultant pediatricians in the Department of Pediatrics for years. The unit runs a robust training in Rheumatology recognized by the National Postgraduate Medical College of Nigeria and the West African College of Physicians.

The Pediatric Rheumatology training program thus formally kick-started in 2018 with a senior registrar in the Department of Pediatrics, posted to the adult rheumatology unit for one year. This was followed by another one-year training in Pediatric Rheumatology at the Pediatric Rheumatology Department of Red Cross War Memorial Children Hospital/University of Cape Town, South Africa. While at Red Cross War Memorial Children's Hospital, she was awarded a clinical and research fellowship in Pediatric Rheumatology at the Alder Hey Children's Hospital/University of Liverpool, Liverpool. Upon completion of this training in February 2022, the Department of Pediatrics formally created a Pediatric Rheumatology Unit, with the approval of the hospital's management and support of the adult Rheumatology Unit.

### Clinical services

The pediatric rheumatology unit offers both inpatient and outpatient specialist rheumatological services to children and adolescents, including procedures such as intra-articular injections. It collaborates with other pediatric specialties in the department to offer these services as necessary. With the set-up of the unit and increasing awareness of its existence among the medical community, for example, via outreach program, the unit is witnessing increasing referrals and diagnoses of children with chronic rheumatological disorders from private and public health facilities. While the majority of the referrals are from within Lagos State and adjoining Ogun State (120 km from Lagos State), some come from as far as Enugu, Anambra, Port Harcourt, and even Abuja (respectively 550, 474, 600, and 760 km from Lagos State).

**Figure 1:**
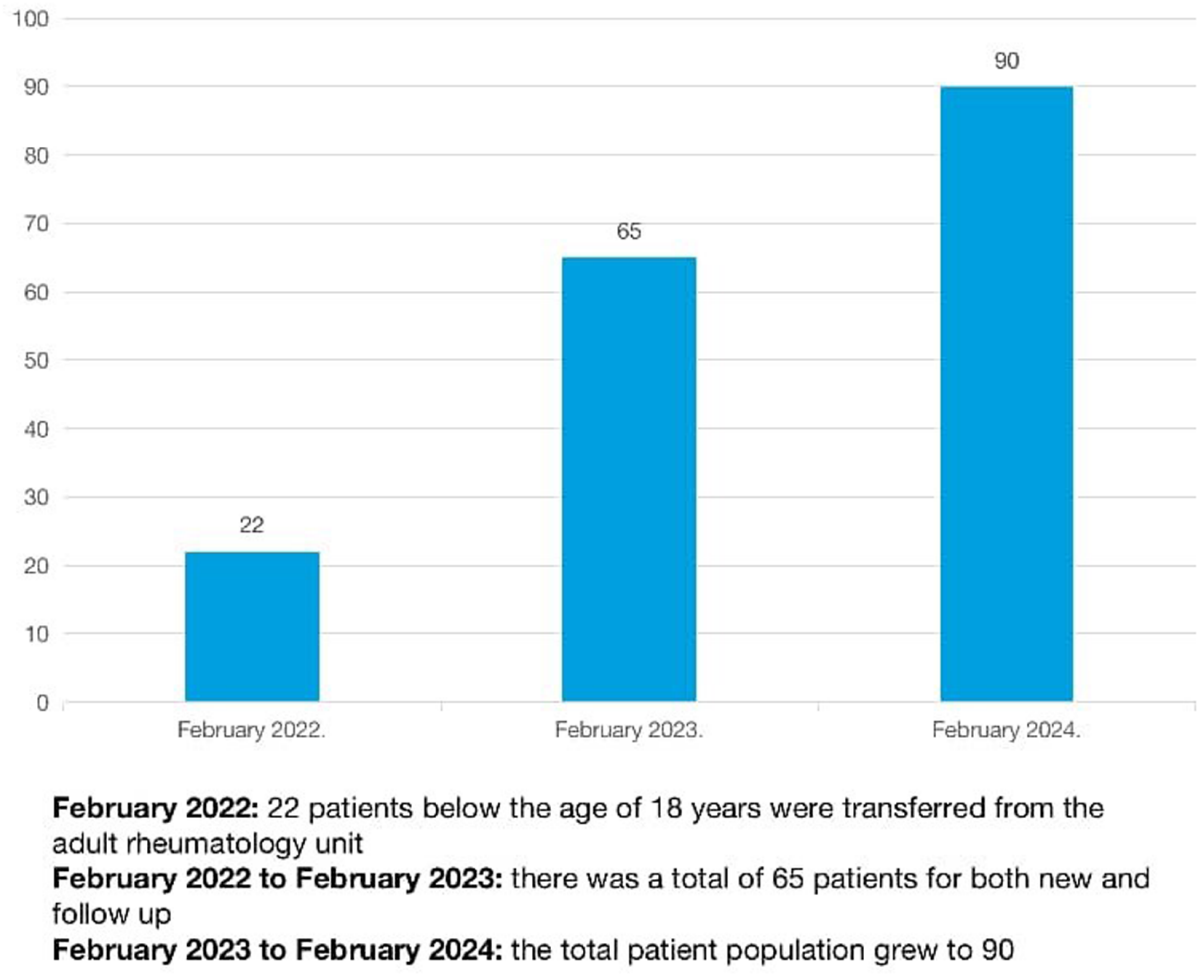
Progression of paediatric rheumatic disease cases in LASUTH.

At the creation of the pediatric rheumatology unit, 22 patients below the age of 18 years were transferred from the adult rheumatology unit. In the first year, from 15th February 2022 to 15th February 2023, there were 65 patients seen for both new and follow-up visits. In the following year (February 15 2023–February 15, 2024), that grew to 90 patients as shown in Figure 1. There were 21 new inpatients, 17 outpatient return visits following discharge, and 46 new outpatient visit.

The spectrum of pediatric rheumatic diseases seen in the first two years of the creation of the clinic is shown in Table 1. Juvenile systemic lupus erythematosus was the most frequent PRD seen in 32 (35.9%) of all cases.

**Table 1:** Spectrum of PRDS in 90 Nigerian children.

Paediatric rheumatic diseases	Frequency (%)
Juvenile idiopathic arthritis	20 (22.5)
Oligoarticular JIA	3 (15)
Polyarticular JIA	9 (45)
Systemic onset JIA	8 (40)
Juvenile connective tissue diseases	
Juvenile systemic lupus erythematosus	32 (35.9)
Juvenile dermatomyositis	3 (3.3)
Polymyositis	3 (3.3)
Juvenile dermatomyositis scleroderma overlap	1 (1.1)
Polyarteritis nodosa	5 (5.6)
Kawasaki disease	3 (3.3)
Systemic sclerosis	1 (1.1)
Granulomatosis with polyangitis	1 (1.1)
Primary Sjogren's syndrome	1 (1.1)
Undifferentiated connective tissue disorder	1 (1.1)
Other rheumatic diseases	
Ankylosing spondylitis	1 (1.1)
Chronic musculoskeletal pain	12 (13.5)
Chronic recurrent multifocal osteomyelitis	1 (1.1)
Fibrodysplasia ossificans progressiva	2 (2.2)
Aquaporin 4 antibodies optic neuritis	1 (1.1)
Multiple sclerosis	1 (1.1)
Hereditary multiple endochondroma	1 (1.1)

### Staffing and training

As part of the Residency Training Program in our Department of Pediatrics, resident doctors (doctors undergoing pediatric specialist training) periodically rotate through the unit, during which they participate in unit clinical and academic activities including ward rounds, bedside teachings, outpatient clinics, and day-ward care. The latter entails parenteral administration of disease-modifying anti-rheumatic drugs like cyclophosphamide and biologics like rituximab, as well as injections of intra-articular steroids. Although currently rheumatology nursing care is provided by the general pediatric nursing staff, it is planned that this would evolve into dedicated pediatric rheumatology nursing care after requisite training. The unit consultant also teaches and trains the medical students whenever the opportunity arises.

## Challenges

Less-resourced countries like Nigeria face a unique set of challenges in caring for children with rheumatic diseases, limiting the ability to deliver high-quality healthcare to these children and their families. These challenges include scarcity of trained pediatric rheumatologists, inadequate healthcare funding mostly as out-of-pocket payments, poor access to laboratory investigations and medications, widespread poverty, low levels of education, and a high burden of chronic infectious and non-infectious disease often mimicking some childhood rheumatological disorders.

### Laboratory support and pharmacotherapy

Inadequate laboratory support is a well-known challenge of healthcare in Nigeria; pediatric rheumatological services are not spared. Laboratory support for rheumatological diagnosis and management still faces significant challenges such as high cost (in the setting of predominantly out-of-pocket healthcare financing), long turnaround time, and non-availability. For serological tests, samples are being sent out of Nigeria, further contributing to delayed diagnosis and expenses.

Similarly, for drugs, most synthetic disease-modifying anti-rheumatic drugs are available with few biologics such as rituximab, etanercept, infliximab, and tocilizumab but are very expensive. While the high cost of medications remains a daunting challenge to optimal treatment for our patients, we continue to advocate with relevant stakeholders to increase access to these life-saving drugs for this vulnerable population. Unfortunately, Nigeria's Health Insurance Scheme currently does not cover serological tests and drugs for rheumatological conditions. Worse still, very few patients have access to health insurance.

### Manpower and training challenges

The absence of rheumatology in the curriculum of medical schools, coupled with the lack of pediatric rheumatology units in postgraduate training centers across the country (apart from LASUTH), possibly contributes to low expertise in the diagnosis of pediatric rheumatic diseases (PRDs) among physicians in the country. Also, there is inadequate funding of non-communicable and rare diseases, including rheumatic conditions (21–23). Presently, to the best of my knowledge, only Lagos State has a pediatric rheumatology service out of the 36 states in Nigeria- this huge gap reflects the scarcity of pediatric rheumatologists across the country, although sometimes filled by adult Rheumatologists where available. According to the Global Burden of Disease data in 2019, the prevalence of musculoskeletal disorders is rapidly increasing, including among children. This calls for urgent and concerted efforts to expand coverage of pediatric rheumatological services through enhanced funding, education, training and enactment of policy.

## Research

Being that pediatric rheumatology is in its infancy in Nigeria, there is an enormous gap in the understanding of the burden and epidemiology of pediatric rheumatic diseases (PRDs) in Nigeria, as well as much of sub-Saharan Africa. There are few case reports/series and single-center descriptive observational studies on PRDs in Nigeria (11–18, 12). For example, Olaosebikan et al. (11) in a retrospective study reported 57 (2.8%) cases of PRDs out of 2,330 cases of rheumatic disease seen at our hospital's adult Rheumatology unit on inpatient and outpatient basis over five years. The pediatric referrals were from the pediatric ward, general pediatric clinic, and peripheral hospitals. Although the study may have possibly under-reported PRDs being from an adult clinic, it provides valuable insight into the burden and spectrum of PRDs. Building on such studies, community-based and prospective cohort studies are now needed to better understand the epidemiology and peculiar behaviors of PRDs in this part of the world. Research support, funding, and infrastructure, as well as international collaboration, will assist local clinicians to conduct such studies in the setting of huge and increasing patient load.

## The future

Albeit the aforementioned challenges, the prospect of this new sub-specialty is bright given appropriate support by critical stakeholders- the government through the Ministry of Health; departmental and institutional heads of undergraduate and postgraduate training schools/colleges; the Pediatric Association of Nigeria; other health professional associations; the pharmaceutical industry; non-governmental organizations; patient support groups; health research funders; the media and the general public. There is a need to continue to raise awareness of pediatric rheumatic diseases (PRDs) among healthcare workers and the community to promote early clinical suspicion, diagnosis, and treatment. Also, there is a need to establish more pediatric rheumatology training centers across the country and include pediatric rheumatology in the medical school curriculum. Federal and State health insurance schemes should be expanded across the country, with the inclusion of rheumatological services in their coverage especially for children. Local, national, and international funders of health research should prioritize funding of basic, epidemiologic, and clinical rheumatologic research. Existing and new pediatric rheumatology training units should foster and build robust international training and research collaborations with more-resourced centers in developed countries for mutual benefit and development. Pediatric rheumatologists who are trained in-country supplemented with robust training and exposure abroad are more likely to better understand and optimize the peculiar behaviors of these diseases in low-resource settings, compared to those trained solely abroad.

## Conclusion

In conclusion, pediatric rheumatology is still a relatively new subspecialty in Nigeria and pediatric rheumatologists are greatly lacking in the country despite its large and growing population. As a result, the healthcare workers must continue to raise awareness of PRDs and ensure prompt referral to the pediatric rheumatologist. The government and relevant stakeholders should ensure the continued existence, growth, and development of the specialty across the country for the betterment of the populace. Now that there is one subspecialty-trained pediatric rheumatologist, hopefully, there can be more ways to grow the field of pediatric rheumatology.

## Data Availability

The raw data supporting the conclusions of this article will be made available by the authors, without undue reservation.
